# *Toxoplasma gondii*: Entry, association, and physiological influence on the central nervous system

**DOI:** 10.1371/journal.ppat.1006351

**Published:** 2017-07-20

**Authors:** Oscar A. Mendez, Anita A. Koshy

**Affiliations:** 1 Graduate Interdisciplinary Program in Neuroscience, University of Arizona, Tucson, Arizona, United States of America; 2 BIO5 Institute, University of Arizona, Tucson, Arizona, United States of America; 3 Department of Immunobiology, University of Arizona, Tucson, Arizona, United States of America; 4 Department of Neurology, University of Arizona, Tucson, Arizona, United States of America; Boston College, UNITED STATES

## Abstract

*Toxoplasma gondii* is one of the world’s most successful parasites, in part because of its ability to infect and persist in most warm-blooded animals. A unique characteristic of *T*. *gondii* is its ability to persist in the central nervous system (CNS) of a variety of hosts, including humans and rodents. How, what, and why *T*. *gondii* encysts in the CNS has been the topic of study for decades. In this review, we will discuss recent work on how *T*. *gondii* is able to traverse the unique barrier surrounding the CNS, what cells of the CNS play host to *T*. *gondii*, and finally, how *T*. *gondii* infection may influence global and cellular physiology of the CNS.

## Introduction

*Toxoplasma gondii* is an obligate intracellular parasite of the phyla Apicomplexa. Felids are the only definitive host for *T*. *gondii*, but *T*. *gondii* has a wide intermediate host range and has been documented to naturally infect most warm-blooded animals including birds, rodents, and humans [1]. In most hosts, *T*. *gondii* establishes a life-long, latent infection in tissues such as skeletal muscle, cardiac muscle, or the central nervous system (CNS), which includes the brain, the spinal cord, and the retina. In this review, we will often use CNS interchangeably with the brain, in which the majority of the work on *T*. *gondii* has been done.

Humans primarily acquire *T*. *gondii* through contaminated food or water or via vertical transmission. *T*. *gondii* is found worldwide and seroprevalence rates range from <10% to >60% [2]. During acute infection, *T*. *gondii* disseminates throughout the host as a tachyzoite, the fast-replicating form of the parasite, which is targeted by the host immune response [3,4]. As infection proceeds, *T*. *gondii* transitions into the chronic stage of infection via conversion to the slowly replicating bradyzoite, which encysts. A number of transitions occur between tachyzoites and bradyzoites/cysts, some of which are thought to enable the bradyzoite/cyst to escape immune detection, thereby leading to persistent infection [5]. In humans and rodents, the brain is the major organ of encystment and persistence [1,6].

The ability of *T*. *gondii* to asymptomatically persist in the CNS of immunocompetent individuals is highly unusual, as when most microbes cross into the CNS, symptomatic and often lethal disease ensues. This tropism for the CNS underlies the devastating disease *T*. *gondii* causes in those with deficient immune responses—e.g., developing fetuses or AIDS patients. Finally, *T*. *gondii*’s predilection for the CNS has been linked to a number of behavioral deficits in rodents [7–11]. Thus, the *T*. *gondii*–CNS interaction is of particular interest for understanding symptomatic toxoplasmosis as well as rodent behavioral changes. As a summary of the behavioral deficits and possible causes has been reviewed recently [12–14], here we will focus on work exploring how *T*. *gondii* enters the CNS, establishes a persistent infection, and affects CNS physiology.

### Brief overview of the CNS

The CNS is a complex organ composed of multiple cells that include neurons and glia. Glia were originally defined as neuronal support cells and consist of oligodendrocytes, astrocytes, and microglia, which are tissue-resident macrophages that have a hematopoietic origin as opposed to neuroectodermal origin [15,16]. What percentage of glia are astrocytes, oligodendrocytes, or microglia will vary by location and state (e.g., baseline versus infected). Neurons are the signaling cells of the CNS and comprise a vast array of subtypes distributed across the CNS in a heterogeneous manner. Regional and long-distance connections between neurons underlie the intricate processing network that allows complex integration of information from external stimuli to initiate and generate movements and complex behaviors. Oligodendrocytes insulate the axons of neurons with myelin, allowing for proper signal conduction. Astrocytes are support units responsible for promoting efficient signaling between neurons, release of growth factors, and separation of neuronal groups [17]. When these cell populations are discussed, the number of glia to neurons is often overestimated. Careful counts suggest that glia and neurons are approximately equivalent in number across a range of mammals, including humans [18–20]. Astrocyte and oligodendrocyte turnover does occur in the CNS, but this turnover becomes attenuated in adulthood, with a vast majority of glia becoming oligodendrocytes [21–23]. For neurons, even in adulthood, replication occurs but only in distinct regions of the brain and in limited amounts [24]. While microglia and infiltrating macrophages are essential for controlling *T*. *gondii* in the brain, neurons and astrocytes are the parenchymal cells that have been most implicated in playing a role in CNS toxoplasmosis.

### Entering the CNS

**Fig 1:**
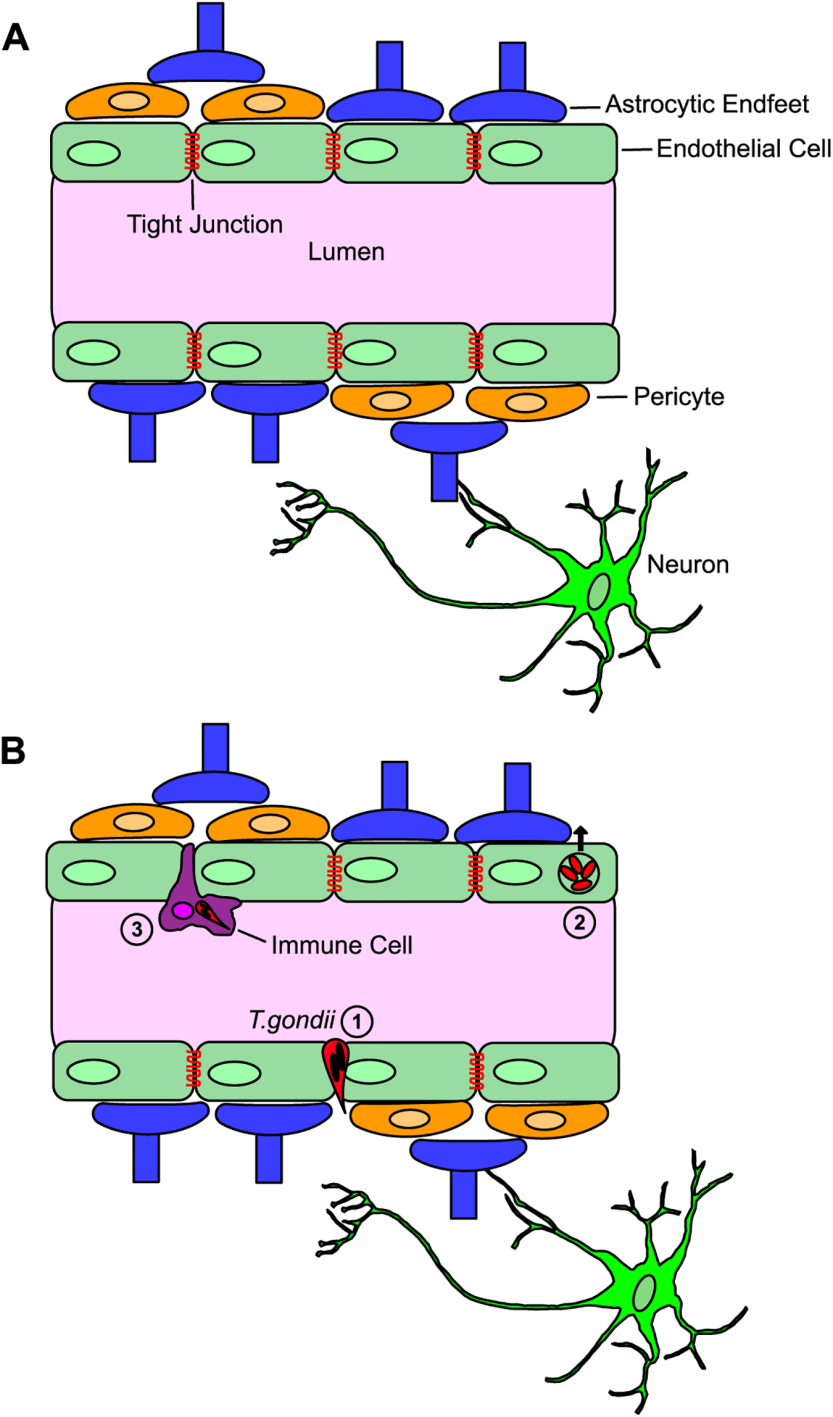
Diagram of the physical and cellular interactions that compose the blood-brain barrier. **(A)** The blood-brain barrier (BBB) is composed of microvessels surrounded by an endothelial cell layer with tight junctions; pericytes surround the tight junctions and then astrocytic processes or endfeet provide the final layer. **(B)** *Toxoplasma gondii* has been postulated to have 3 mechanisms for crossing the BBB. **(1)** Paracellular entry, in which *T*. *gondii* migrates directly through the tight junctions of the endothelial cell layer, **(2)** transcellular entry, in which free parasites in the vascular compartment infect endothelial cells, replicate, and then egress out of the basolateral side of endothelial cell (lysing the host cell), **(3)** the “Trojan horse” method, whereby an infected immune cell infiltrates the CNS, after which the parasite egresses out of the immune cell and into the brain parenchyma.

For any pathogen to enter the CNS, it must cross the blood-brain barrier (BBB). The BBB is a selective barrier that is composed of endothelial cells that line microvessels in the brain. The basal lamina and pericytes surround endothelial cells, followed by enclosing astrocytic endfeet (Fig 1A). These cellular interactions allow the BBB to exclude large peptides and proteins and only allow free diffusion of small gaseous molecules like oxygen and carbon dioxide. At baseline, the presence of tight junctions and adherence junctions excludes paracellular movement of hydrophilic molecules and the migration of cells past the endothelial barrier [25]. Though originally conceived of as a highly impermeable barrier through which most organisms and cells could not pass, it is now recognized that microbes have developed multiple mechanisms for crossing the BBB, and in the right context, immune cells cross into the CNS for immune surveillance [26–29]. For *T*. *gondii*, 3 mechanisms have been proposed for CNS entry: paracellular crossing, transcellular crossing, or the so-called “Trojan horse” mechanism, whereby an infected immune cell crosses the BBB, bringing the intracellular parasite with it (Fig 1B).

### Paracellular entry

Several lines of evidence suggest that *T*. *gondii* could cross into the CNS via the paracellular method (Fig 1B, part 1). Though *T*. *gondii* lacks cilia and flagella, the parasite is able to propel itself using actin-myosin motors, generating movement termed “gliding motility” [30]. This gliding motility is thought to aid *T*. *gondii* across the first barrier it encounters: the epithelium of the small intestine [31]. The gut epithelial barrier shares many features with the BBB including tight junctions, paracellular junctions, regulation of barrier properties, and an immune barrier [32,33]. Parasites are able to cross polarized cell monolayers and extracellular matrix, which mimic both the BBB and gut epithelial barrier [34]. In addition, parasites are able to transmigrate across the intestinal epithelium *ex vivo* [34]. Importantly, recent work using physiological shear force applied to live-cell microfluidic chambers has shown that tachyzoites are capable of adhering to and migrating on vascular endothelium in these semiphysiologic conditions [35], though actual paracellular crossing by tachyzoites was not observed in these conditions (personal communication, M. Lodoen to A. Koshy).

### Transcellular migration

While *Plasmodium* sporozoites have been observed to migrate through cells [31], for *T*. *gondii*, transcellular migration refers to infection of a cell followed by replication then lyses or egress from the basolateral side (Fig 1B, part 2). This concept has been primarily conceptualized for crossing from the gut epithelium into the intestinal lamina propria [36,37]. However, a recent study utilizing transgenic reporter systems and multiphoton in vivo imaging suggests such a mechanism may also have a role in how *T*. *gondii* crosses the BBB. In this study, Konradt et al. found infected endothelial cells in multiple organs including the brain. Further work showed that free tachyzoites in the bloodstream were able to adhere to CNS endothelial cells, invade, replicate, and eventually egress from these cells, potentially depositing these egressing parasites into the CNS parenchyma [38]. This work also used in vitro assays to show that, in shear stress conditions, parasites were able to attach and invade endothelial cells, especially at lower shear forces, consistent with the in vivo observation that parasite-infected endothelial cells were primarily found in smaller diameter vessels [38]. Finally, this study noted a lack of infected infiltrating immune cells early in CNS infection, consistent with prior work that also noted CNS parasite infection preceding immune-cell infiltration into the CNS [39].

### Infected immune cells

The final mechanism proposed for *T*. *gondii* entry into the CNS is via the “Trojan horse” mechanism (infected immune cells) (Fig 1B, part 3). Several studies using in vitro models have shown that infected immune cells have increased motility and are capable of crossing endothelial barriers, including during shear stress [40–42]. Additionally, intravenous inoculation of mice with infected macrophages or dendritic cells led to a more rapid appearance of parasites in the CNS compared to inoculation with free parasites [40,43], though multiple mechanisms (not just increased BBB crossing by infected immune cells) might explain these results.

In summary, *T*. *gondii* may enter the CNS through multiple mechanisms. Outstanding questions include the relative importance of each mode of entry and how different mechanisms of entry might affect which regions of the CNS are infected or even which CNS cells directly interact with *T*. *gondii*.

## CNS host cell interactions

Given the importance of CNS persistence to clinical disease, where and how *T*. *gondii* persists in the CNS in the immunocompetent host has long been an area of interest. Human data on CNS regions “susceptible” to *T*. *gondii* primarily come from autopsies of AIDS patients. In these studies of severely immunocompromised patients, *T*. *gondii* lesions were consistently found in the cerebral cortex and basal ganglia, with fewer lesions in the cerebellum, brainstem, and spinal cord [44–46]. These data are consistent with the localization of cysts observed in rodents [47–49].

Host cell–*T*. *gondii* interactions of immunocompetent humans are not well characterized. One recent report from primarily immunocompetent patients states that *T*. *gondii* was seen in both neurons and astrocytes, but it is unclear from the methodology how this observation was determined [50]. Given the lack of human data, our understanding of CNS host cell–*T*. *gondii* interactions has, by necessity, come from in vitro and rodent models of CNS toxoplasmosis. In mouse models of toxoplasmosis, some controversy existed over whether *T*. *gondii* cysts were intra- or extracellular [51,52], but the earliest reports using electron microscopy (EM) suggested that cysts were within cells, though this work did not identify the specific cell type [53,54]. Subsequent improvements in EM led to the identification of synapses near cysts, suggesting that cysts persisted within neurons [55]. The finding that cysts are intracellular and primarily within neurons was further supported by additional studies, including 1 that utilized parasites recently isolated from AIDS patients and a common lab-passaged strain [56,57]. A recent study utilizing immunofluorescence and confocal microscopy also found that cysts were primarily associated with antineuronal labeling [58].

The recognition that *T*. *gondii* persists primarily in neurons raised the question of why *T*. *gondii*—a parasite that can invade most nucleated cells in vitro [59] and that brings its own invasion machinery [60]—predominantly persists in neurons. In vitro studies showed that *T*. *gondii* readily infects and encysts in both astrocytes and neurons [61,62], but astrocytes were capable of using multiple mechanisms to clear intracellular parasites [63–66]. Conversely, neurons were not capable of such clearance [67], a finding in line with other evidence that neurons lacked full immune-response capabilities [68]. Based upon these studies and the previously reviewed in vivo work, the working model for *T*. *gondii*–CNS host cell interactions was that after entering the CNS, parasites invaded both astrocytes and neurons. Astrocytes then cleared the intracellular parasites while neurons could not, leaving neurons as the host cell for persistent infection.

**Fig 2:**
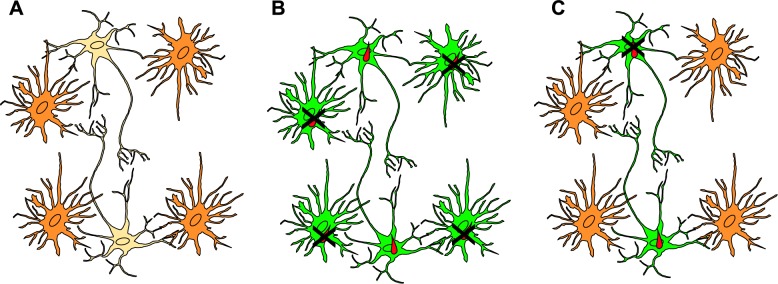
Using the *Toxoplasma gondii*–Cre system to test 2 models of why *T*. *gondii* primarily persists in neurons in vivo. **(A)** After entering the CNS, *T*. *gondii* should be able to interact with different cells in the brain, including both astrocytes (orange) and neurons (beige). As the *T*. *gondii*–Cre system leads to the expression of a green fluorescent protein (GFP) in host cells injected with *T*. *gondii* protein regardless of infection status, it can help distinguish between 2 likely models for *T*. *gondii* persistence in neurons. **(B)** Model 1: after infiltration of the CNS, *T*. *gondii* interacts with and invades both astrocytes and neurons, causing both cell types to express GFP (green). Astrocytes clear the intracellular parasite while neurons cannot, leaving neurons as the primary host cell for persistent infection. **(C)** Model 2: after infiltration of the CNS, *T*. *gondii* almost exclusively interacts with and invades neurons, leading to GFP expression primarily in neurons. Neurons potentially clear some but not all invading parasites, leaving neurons as the primary host cell for persistent infection.

Until recently, this model could not be tested in vivo as there was no way to identify host cells that had been invaded but cleared the intracellular parasite. The advent of the *T*. *gondii*–Cre system, an in vivo system in which parasites trigger permanent host-cell expression of green fluorescent protein (GFP) even when the cell is not productively infected, offered a platform to test this model (Fig 2) [69,70].

Studies using this system suggest that (1) *T*. *gondii* interacts with far more CNS cells than previously described, (2) the majority of these interactions do not lead to productive infection, and (3) throughout CNS infection, *T*. *gondii* predominantly and almost exclusively interacts with neurons [70,71]. These data suggest a new model for *T*. *gondii*–CNS host cells, one in which *T*. *gondii* persists in neurons because parasites primarily interact with and invade neurons.

Many outstanding questions remain, which include (1) determining what factors drive the *T*. *gondii*–neuron interaction, (2) if the injected but uninfected neurons arise through aborted invasion and/or neuron clearance of intracellular parasites, and (3) how uninfected, injected neurons differ from (or are the same as) infected neurons, especially in terms of neuroanatomic localization, gene expression, and electrophysiology.

## Physiological changes and effects during *T*. *gondii* infection

Until recently, little work had been done on how *T*. *gondii* changes CNS physiology, but in the last several years, a number of important in vivo studies have begun to address this question. A major mechanism for affecting CNS physiology would be through changes in neurotransmitters, the molecules that neurons use for interneuronal communication. In vivo measurements of neurotransmitters have primarily measured global changes within the brain, not cell-specific changes. The dopaminergic system has been of major interest because dopamine is essential for locomotor activity (movement) and various forms of learning, including fear [72]. As such, investigators have sought to implicate the dopaminergic system in infection-induced behavioral changes [9]. Several studies that directly measured CNS dopamine levels or dopamine metabolites suggest that postinfection, CNS dopamine levels increase [73,74], though another group was unable to confirm these changes [75]. One explanation for these contradictory findings is that each group used different mouse strains, which can affect the immune response to *T*. *gondii* [76,77]. As immune cells have been shown to make dopamine [78] and none of the groups determined the cellular source of the measured dopamine or metabolites, these differences might simply reflect differing levels of immune infiltration into the CNS rather than changes in the CNS dopaminergic cells or pathways.

**Fig 3:**
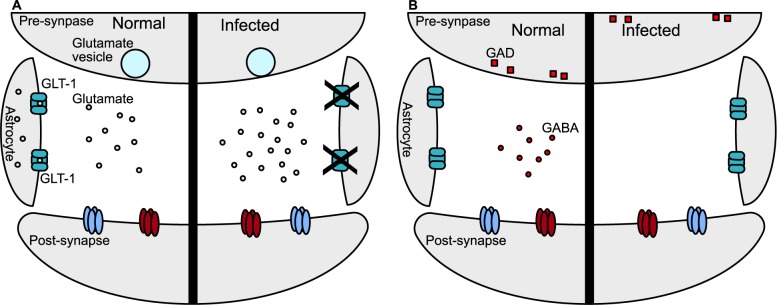
Schematic of *Toxoplasma gondii* effects on glutamate and glutamate decarboxylase. **(A)** Under normal conditions, glutamate, an excitatory neurotransmitter, is released into the synaptic cleft from the presynaptic neuron. Glutamate then diffuses across the synaptic cleft to act on glutamate receptors on the postsynaptic neuron, leading to excitation of the postsynaptic neuron. This glutamatergic signaling is terminated by uptake and recycling of synaptic glutamate by the glutamate transporter GLT-1 on surrounding astrocytes. Glutamate uptake by GLT-1 is essential to avoid excessive glutamate signaling, which can lead to postsynaptic excitotoxicity and neuronal death. After an infection, GLT-1–dependent transport into astrocytes is impaired, allowing for an increase in glutamate accumulation in the synaptic cleft, which is expected to lead to more excitation of the postsynaptic neuron. **(B)** Glutamate decarboxylase (GAD) is primarily found on presynaptic terminals, where it will process glutamate into γ-aminobutyric acid (GABA), the major inhibitory neurotransmitter in the brain. Once GABA is released into the synaptic cleft, it will bind onto GABA receptors on the postsynaptic neuron, which decreases the excitability of the postsynaptic neuron. In the case of infection, GAD is redistributed into the cytosol of the presynaptic neuron, which would be expected to cause improper GABA localization at synapses, leading to decreased inhibition of the postsynaptic neuron.

Two recent papers have focused on the neuron neurotransmitter–physiology link and collectively found that the infected CNS shows altered excitability. David et al. found that glutamate, the major excitatory neurotransmitter of the CNS, was increased in the CNS of infected mice secondary to disruption of the normal reuptake of extracellular glutamate by astrocytes via the glutamate transporter (GLT-1) (Fig 3). They further showed that these changes were correlated with decreases in neuronal dendritic spine density (an early sign of distressed neurons) as well as depth-electrode electroencephalogram (EEG) changes and that most of these changes could be reversed with a brief treatment with Ceftriaxone, an antibiotic that increases GLT-1 levels [79]. Brooks et al. examined the effect of *T*. *gondii* infection on γ-aminobutyric acid (GABA), the major inhibitory neurotransmitter in the brain. Within the hippocampus, they showed that glutamate decarboxylase (GAD), a key enzyme responsible for the conversion of glutamate to GABA and the most common marker for identifying GABAergic neurons, was mislocalized within neurons in this region, though the absolute levels of GAD were unchanged compared to uninfected mice. Though the authors did not directly measure GABA levels, they did use skull EEG recordings to show that infected mice had an increase in spontaneous seizures and sensitivity to drug-induced seizures, both of which would be expected consequences of the loss of neuronal inhibition [80]. In addition to effects on neurotransmitters, infection has been noted to cause physical changes to the CNS. One study that evaluated mice infected with *T*. *gondii* for 8 months found that infected mice had both an increase in the size of the ventricles, fluid-filled spaces in the center of the brain, as well as some asymmetry of the brain parenchyma. While both findings could be explained by loss of parenchymal cells in the brain, histologic studies showed no neuronal loss, axonal injury, or “extensive” demyelination [11]. However, another study using diffusion tensor imaging, an MRI method used to evaluate white matter projections (the axons of neurons and the oligodendrocytes that produce the myelin that ensheaths axons) found mistargeting of discrete neuronal projections in mice chronically infected for 4–5 months compared to control animals. This study specifically focused on the somatosensory cortex (SSC), the part of the brain involved in tactile modes of sensation, as the authors had noted abnormalities in the SSC in full brain evaluations. Following up with immunofluorescence and Western blots, the authors observed reductions in dendritic arborization, spine number, and essential synaptic proteins, suggesting that the synaptic connections of the SSC had been disrupted [81]. This latter study suggests that *T*. *gondii* infection causes discrete areas of disruption and neuronal loss, which may be missed with global surveys rather than focal studies.

In summary, what has become apparent is that the form and physiology of the rodent brain are affected during both acute and chronic infection. The mechanisms that cause these changes in CNS physiology may be due to (1) direct parasite interactions via injection of effector proteins or persistence within a host neuron, (2) indirect effects of the CNS immune response to control the infection, or (3) both. Additionally, as these studies focused on relatively acute infections, the duration of these changes has yet to be determined. In addition, it remains unclear how these findings translate to human outcomes, though both congenitally infected patients and AIDS patients with toxoplasmic encephalitis are known to have seizures [82,83].

## Conclusion

In the last decade, important work has been done to better define many molecular and cellular aspects of the *T*. *gondii*–CNS interaction. While these studies leave a number of outstanding questions as noted above, they form the foundation of an exciting time in the evolution of our understanding of CNS toxoplasmosis.
